# Adiponectin Expression in the Porcine Ovary during the Oestrous Cycle and Its Effect on Ovarian Steroidogenesis

**DOI:** 10.1155/2014/957076

**Published:** 2014-03-26

**Authors:** Anna Maleszka, Nina Smolinska, Anna Nitkiewicz, Marta Kiezun, Katarzyna Chojnowska, Kamil Dobrzyn, Hubert Szwaczek, Tadeusz Kaminski

**Affiliations:** Department of Animal Physiology, University of Warmia and Mazury in Olsztyn, Ulica Oczapowskiego 1A, Kortowo, 10-719 Olsztyn, Poland

## Abstract

Adiponectin is an adipose-secreted hormone that regulates energy homeostasis and is also involved in the control of the reproductive system. The goal of the present study was to investigate changes in adiponectin gene and protein expression in porcine ovarian structures during the oestrous cycle and to examine the effects of *in vitro* administration of adiponectin on basal and gonadotrophin- and/or insulin-induced secretion of ovarian steroid hormones. Both gene and protein expression of adiponectin were enhanced during the luteal phase of the cycle. Adiponectin affected basal secretion of progesterone by luteal cells, oestradiol by granulosa cells, and testosterone by theca interna cells. The gonadotrophin/insulin-induced release of progesterone from granulosa and theca interna cells and the release of oestradiol and androstenedione from theca cells was also modified by adiponectin. In conclusion, the presence of adiponectin mRNA and protein in the porcine ovary coupled with our previous results indicating adiponectin receptors expression suggest that adiponectin may locally affect ovarian functions. The changes in adiponectin expression throughout the oestrous cycle seem to be dependent on the hormonal status of pigs related to the stage of the oestrous cycle. The effect of adiponectin on ovarian steroidogenesis suggests that this adipokine influences reproductive functions in pigs.

## 1. Introduction

The adipose tissue is an endocrine organ that produces various factors including the adipokines [1]. One of them, adiponectin, also known as Acrp30, AdipoQ, GBP28, and apM1, was discovered by four independent research teams in 1995/1996, [2–5] as the most abundant product of the adipose tissue [6]. The hormone is a 244-amino acid protein with 30 kDA molecular weight that circulates in the serum in different multimeric forms [5]. Actions of adiponectin are mediated by two types of receptors: adiponectin receptor 1 (AdipoR1) and 2 (AdipoR2). The receptors are highly related and share 67% sequence identity in mice, but they differ in their tissue distribution and the ability to bind various forms of adiponectin. AdipoR1 shows high affinity for the globular form of adiponectin, whereas AdipoR2 is characterized by intermediate binding affinity for both globular and full-length adiponectin [7]. The highest levels of AdipoR1 expression are observed in skeletal muscles, whereas AdipoR2 is most highly expressed in the liver [7, 8]. Due to the extensive distribution of adiponectin receptors in peripheral tissues and organs, adiponectin exerts pleiotropic effects on metabolism, including regulation of homeostasis by fatty acid oxidation, stimulation of glucose uptake and gluconeogenesis inhibition, which leads to intensified thermogenesis and weight loss [9]. Consequently, adiponectin level correlates negatively with body fat [10] and positively with insulin sensitivity [11].

The existing evidence points to the presence of a common endocrine system comprising several hormones, including ghrelin, leptin, and orexin that controls metabolism and reproductive functions [12–15]. It is believed that adiponectin belongs to the above group of hormones. Adiponectin mRNA and protein were found in the ovaries of several species, including humans, rats, and cows [16–19]. Adiponectin mRNA and protein were also detected in the ovaries of prepubertal but not sexually mature gilts [8]. The influence of the hormonal status of swine related to the stage of the oestrous cycle on adiponectin expression in corpora lutea, granulosa, and theca interna cells remains unknown. Studies indicating higher concentrations of adiponectin in female than in male blood suggest that ovarian steroids may regulate adiponectin secretion [20, 21]. Similar conclusions can be drawn from varied plasma levels of adiponectin during the oestrous cycle in pigs [22]. Despite the above, the possible influence of adiponectin on the production of steroid hormones by porcine ovarian cells remains unexplored. Therefore, the aim of this study was to investigate the expression of the adiponectin gene by real-time PCR, to determine the concentrations of adiponectin protein in the porcine ovary (corpora lutea, granulosa and theca interna cells) and to compare gene and protein expression levels during different stages of the oestrous cycle in pigs. Additionally, we sought to determine the* in vitro* effect of adiponectin on the secretion of steroid hormones (progesterone, oestradiol, testosterone, and androstenedione) by ovarian luteal, granulosa, and theca interna cells of sexually mature swine.

## 2. Materials and Methods

### 2.1. Experimental Animals

All experiments were carried out in observance of the ethical standards of the Animal Ethics Committee at the University of Warmia and Mazury in Olsztyn. The experimental material comprised mature gilts (Large White and Polish Landrace) from a private breeding farm, aged 7-8 months and weighing 130–140 kg. Twenty gilts were divided into four experimental groups as follows: days 2-3, 10–12, 14–16, and 17–19 of the oestrous cycle. Females were monitored daily for oestrus behaviour in the presence of an intact boar. The day of the second oestrus was designated as day 0 of the oestrous cycle. The phase of the oestrous cycle was also determined based on ovarian morphology [23]. Additionally, to fully confirm correctness of the evaluation of the oestrous cycle phase, the level of progesterone was determined [24]. The ovaries were removed and placed on ice within minutes after slaughter. Dissected corpora lutea (CLs) from different stages of the oestrous cycle (days 2-3-corpora haemorrhagica, 10–12-mature CLs, and 14–16-regressing CLs) were either immediately frozen in liquid nitrogen and stored at −80°C until RNA and protein analysis or, similarly to the ovaries from days 17–19 of the cycle, placed in cold PBS buffer and transported to the laboratory where luteal, follicular granulosa, and theca interna cells were isolated. Granulosa and theca interna cells are precursor cells of large and small luteal cells, respectively.

### 2.2. Isolation of Luteal, Granulosa, and Theca Interna Cells

Luteal cells were isolated by the method described by Kaminski et al. [25]. Dissected corpora lutea from ovaries on days 2-3, 10–12, and 14–16 were minced into small fragments and dispersed in F-12 medium containing bovine serum albumin fraction V (BSA; 1%) and antibiotics. Corpora lutea were enzymatically dissociated in 0.125% trypsin solution (4–6 times) at 37°C, centrifuged (300 ×g, 10 min, 4°C), and washed three times. Isolated luteal cells were filtered through nylon mesh (40 *μ*m in diameter) and resuspended in fresh F-12 medium. The cells were counted using a haemocytometer, and their viability (~90%) was determined by 0.4% trypan blue dye exclusion.

Granulosa and theca interna cells were isolated from large follicles (diameter > 6 mm) without signs of atresia. Granulosa cells were aspirated with a syringe and additionally washed out with a strong stream of media directed to the internal wall of the follicle [26]. The theca interna layer was scraped off from granulosa cells and enzymatically dispersed in 0.25% trypsin solution [27]. Dispersed cells were centrifuged (800 ×g for 10 min) and washed two and three times to isolate granulosa and theca interna cells, respectively. The cells were filtered through nylon mesh (40 *μ*m in diameter) and resuspended in Eagle's medium enriched with BSA (5%) and antibiotics. The cells were counted using a haemocytometer, and their viability (~98%) was determined by 0.4% trypan blue dye exclusion. Some of granulosa and theca interna cells (5 × 10^6^ viable cells within each experiment) were immediately resuspended in TRIzol reagent (Invitrogen, USA) and stored at −80°C until processing for RNA analysis.

### 2.3. Total RNA Isolation, cDNA Synthesis and Quantitative Real-Time PCR

Total RNA was extracted from luteal tissues from days 2-3, 10–12, and 14–16 of the oestrous cycle using the Absolutely RNA Miniprep Kit (Stratagene, USA). In the case of granulosa and theca cells total RNA was extracted using the TRIzol reagent. RNA concentration and quality were determined spectrophotometrically (NanoDrop ND-1000, NanoDrop Technologies Inc., USA). Approximately 1 *μ*g of RNA was reverse-transcribed into cDNA in a total volume of 20 *μ*L with 0.5 *μ*g oligo (dt)_15_ primer (Roche, Germany) using the Omniscript RT Kit (Qiagen, USA) at 37°C for one hour and was terminated by incubation at 93°C for 5 min. Quantitative real-time PCR analysis was performed using a PCR System 7300 (Applied Biosystems, USA). Sense and antisense primers (adiponectin, cyclophilin A) were chosen according to Lord et al. [8] study. Forward and reverse primers for glyceraldehyde (GAPDH) were used according to Nitkiewicz et al. paper [24]. The chosen primers were as follows: adiponectin forward: 5′ATGATGTCACCACTGGCAAATTC-3′, reverse: 5′-GACCGTGACGTGGAAGGAGA-3′; cyclophilin A, forward: 5′-GCACTGGTGGCAAGTCCAT-3′, reverse: 5′-AGGACCCGTATGCTTCAGGA-3′; GAPDH forward: 5′-CCTTCATTGACCTCCACTACATGGT-3′, reverse: 5′-CCACAACATACGTAGCACCAGCATC-3′. Adiponectin primers (access no: AY135647) were complementary to positions 514–536 (F) and 565–584 (R) of pig adiponectin gene sequence; cyclophilin A primers (access no: AY266299) were complementary to positions 219–237 (F) and 269–299 (R) of pig cyclophilin A gene sequence, GAPDH primers (access no: U48832) were complementary to positions 61–85 (F) and 219–243 (R) of pig GAPDH. A constitutively expressed genes, cyclophilin A and GAPDH, were used as the internal control to verify the quantitative real-time PCR. To ensure that cyclophilin A and GAPDH were the suitable reference genes for this study, we revealed that there were no statistically significant differences in Ct values between the examined ovarian structures throughout all investigated stages of the oestrous cycle. Both of the housekeeping genes were also indicated as suitable reference genes (internal controls) in the independent studies [28]. The PCR reaction included 20 ng cDNA, 900 nM (adiponectin forward), 300 nM (adiponectin reverse, cyclophilin A forward and reverse) and 60 nM (GAPDH forward and reverse) primers, 12.5 *μ*L SYBR Green PCR Master Mix (Applied Biosystems, USA), and RNase free water in a final volume of 25 *μ*L. Quantitative real-time PCR cycling conditions were as follows: initial denaturation and enzyme activation at 95°C for 10 min, followed by 40 cycles of denaturation at 95°C for 15 s and annealing at 59° for 1 min. Negative controls were performed in which water was substituted for cDNA, or reverse transcription was not performed prior to PCR. All samples were amplified in duplicate. The specificity of amplification was tested at the end of the PCR by melting-curve analysis. Product purity was confirmed by electrophoresis. Calculation of relative expression levels of adiponectin was conducted based on the comparative cycle threshold method (ΔΔCt) [29] and normalized using the geometrical mean of reference genes expression levels: GAPDH and cyclophilin A.

### 2.4. Western Blotting

Western blotting analysis was performed as described by Smolinska et al. [30]. Briefly, equal amounts of porcine corpora lutea as well as granulosa and theca cells lysats (10 *μ*g) were resolved by SDS-PAGE on 12.5% polyacrylamide gel and then transferred to a nitrocellulose membranes (Whatman, USA). Blots were blocked for 5 h at 4°C in 1 × TBST containing 5% skimmed milk powder and then incubated overnight at 4°C with the rabbit polyclonal adiponectin antibodies at the dilution of 1 : 150 (Santa Cruz Biotechnology, USA) or rabbit polyclonal actin antibodies (Sigma, USA) diluted 1 : 200, which were used as an internal control for equal loading and to quantify porcine adiponectin protein. To identify immunoreactive bands, membranes were incubated for 1.5 h at room temperature with mouse anti-rabbit IgG for adiponectin (Sigma, USA; diluted 1 : 2000), goat anti-rabbit IgG for actin (diluted 1 : 5000) conjugated with alkaline phosphatase (Santa Cruz Biotechnology, USA). Nonspecific foetal calf serum (MP Biomedicals, USA) was used instead of primary antibodies to produce negative control blots. The immunocomplexes were visualized using 4-nitroblue tetrazolium chloride (NBT) and 5-bromo-4-chloro-3-indolyl phosphate (BCIP), according to the manufacture's protocol (Promega, USA). The results of Western blotting were quantified by densitometric scanning of immunoblots with GelScan for Windows ver. 1.45 software (Kucharczyk, Poland). Data were expressed as a ratio of adiponectin protein relative to actin protein in arbitrary optical density units.

### 2.5. *In Vitro* Cultures of Luteal, Granulosa, and Theca Interna Cells

Luteal cells (250000/1 mL medium) were resuspended in F-12 medium enriched with foetal calf serum (FCS; 20%), BSA (1%) and antibiotics and preincubated for 48 hours in a humidified incubator with 95% air and 5% CO_2_ atmosphere. The serum-containing medium was discarded, and the cells were washed using serum-free F-12 medium. After washing, luteal cells were cultured for 24 hours in F-12 medium with BSA (1%) and antibiotics, with or without treatment agents.

Granulosa and theca interna cells (250000/1 mL medium) were resuspended in Eagle's medium supplemented with 10% FCS, 5% BSA, and antibiotics. After 24 hours of preincubation, the medium was discarded, and the cells were washed and cultured for 24 hours in Eagle's medium with 5% BSA and antibiotics, with or without treatment reagents.

The cultured cells were treated with 1 or 10 *μ*g/mL of recombinant human adiponectin (Biovendor, USA) alone or in combination with insulin (granulosa and theca interna cells; 10 ng/mL; Sigma, USA), FSH (granulosa cells; 10 ng/mL; NHPP, USA) and LH (theca interna cells; 10 ng/mL; NHPP, USA), and combination of those treatments (insulin + FSH for granulosa cells and insulin + LH for theca interna cells). Adiponectin doses used in the experiment were close to physiological concentrations of this hormone in the porcine blood [22]. Luteal cells were cultured only with adiponectin (both doses) without other stimulators. Those cells are thought to be generally more autonomic than follicular cells which is a reason of luteal cells' hard response to stimulators [31–33]. The Alamar blue test revealed that none of the treatments affected the viability of the cultured cells. All incubations were performed in duplicate. Following incubation, the media were harvested, centrifuged (800 ×g for 10 min), and the supernatants were collected and stored at −20°C until analyses of progesterone (cultures of luteal, theca interna, and granulosa cells), oestradiol (cultures of theca interna and granulosa cells), androstenedione, and testosterone (cultures of theca interna cells).

#### 2.5.1. Radioimmunoassays of Steroid Hormones

Progesterone (P_4_) was analysed according to the method described by Ciereszko et al. [34], oestradiol (E_2_) was determined according to the method of Hotchkiss et al. [35] modified by Kotwica [36], androstenedione (A_4_) as described by Dziadkowiec et al. [37], and testosterone (T) according to Kotwica and Williams [38]. Cross-reactivities of the antiserum against P_4_ have been published previously [37]. The specificity of the antibodies against A_4_, T, and E_2_ has been reported by Ciereszko et al. [39] and Szafranska et al. [40]. The sensitivities of the assays for P_4_ and A_4 _were 1 pg/mL and for E_2_ and T were 0.5 pg/mL. Intra- and interassay coefficients of variation of the P_4_, E_2_, A_4,_ and T assays were 0.91%, 0.7%, 0.81%, 0.97% and 8.5%, 10.1%, 14.2%, and 7.8%, respectively.

### 2.6. Statistical Analysis

Data from real-time analysis and Western blot were analysed by one-way ANOVA and least significant difference (LSD) post hoc test and are reported as the means ± SEM from five independent observations. All data concerning* in vitro* cell cultures were analysed by ANOVA for repeated measurements and least significant difference (LSD) post hoc test and are reported as the means ± SEM from five (for all types of cells) independent observations. Each experiment was performed on different day using separate pool of luteal or follicular cells. Statistical analysis was performed using the Statistica program (StatSoft Inc., Tulsa, USA). Values for *P* < 0.05 were considered statistically significant.

## 3. Results

### 3.1. Adiponectin Gene Expression

**Figure 1:**
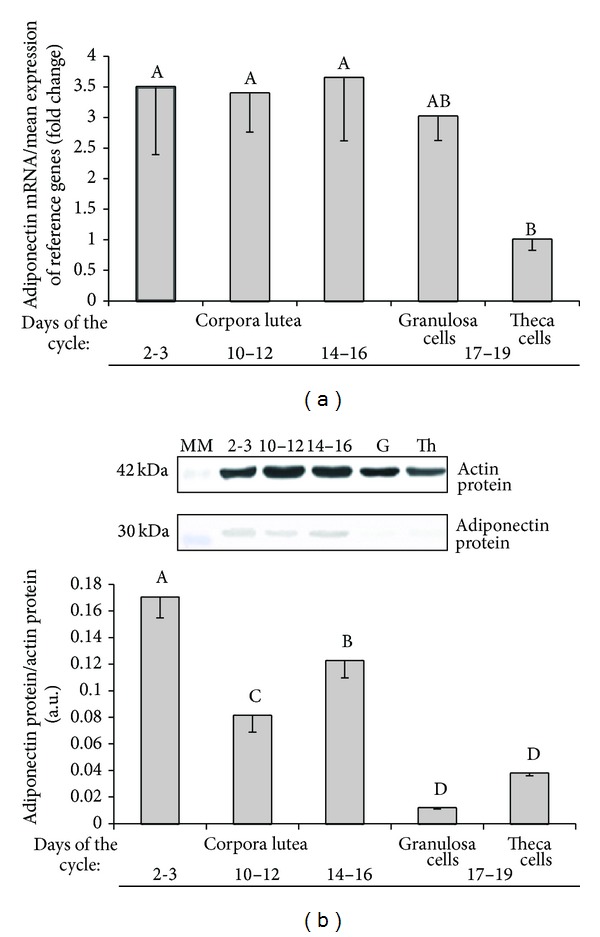
(a) Adiponectin mRNA expression determined by quantitative real-time PCR in porcine corpora lutea, granulosa cells, and theca interna cells harvested on days 2-3, 10–12, 14–16, and 17–19 of the oestrous cycle. The results are expressed as means ± S.E.M. (*n* = 5). Bars with different letters at the top are different (*P* < 0.05). (b) Adiponectin protein expression determined by Western blotting analysis in porcine corpora lutea, granulosa, and theca interna cells on days 2-3, 10–12, 14–16, and 17–19 of the oestrous cycle. Upper panel: representative immunoblots (MM—molecular marker, G—granulosa cells, Th—theca interna cells); lower panel: densitometric analysis of adiponectin protein relative to actin protein. The noted values are expressed as means ± S.E.M. in arbitrary optical density units (*n* = 5). Bars with different letters at the top are different (*P* < 0.01).

The expression of adiponectin mRNA was significantly higher in corpora lutea during all investigated days of the luteal phase than in theca interna cells isolated on days 17–19 of the cycle (*P* < 0.05). No significant differences were noted in adiponectin gene expression in granulosa cells compared with theca interna cells and CLs (Figure 1(a)).

### 3.2. Concentration of Adiponectin Protein

In general, adiponectin protein concentration in the porcine ovary was higher in the luteal phase than in the follicular phase. In particular, the adiponectin protein content was the greatest in CLs from days 2-3 in comparison with CLs and follicular cells from the remaining stages of the oestrous cycle (*P* < 0.01). Within the luteal phase adiponectin concentration was the lowest in CLs during days 10–12 (*P* < 0.01). There was no significant differences in protein expression between granulosa and theca interna cells (Figure 1(b)).

### 3.3. Effects of Adiponectin on Progesterone Secretion by Luteal Cells

**Figure 2:**
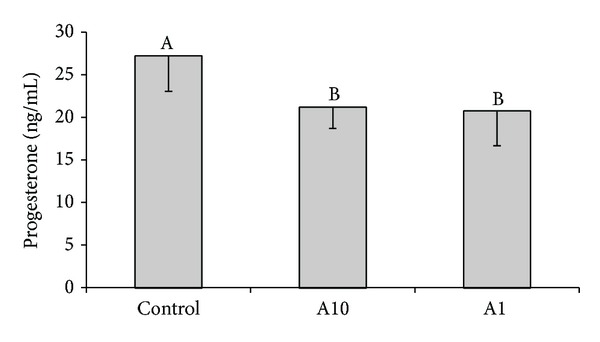
The effect of adiponectin (10 *μ*g/mL-A10 and 1 *μ*g/mL-A1) on basal progesterone secretion by cultured luteal cells collected on days 10–12 of the oestrous cycle. Bars with different superscripts are significantly different (*P* < 0.05). Result are means ± S.E.M. of five replicates.

Treatment of luteal cells from days 10–12 of the oestrous cycle with adiponectin at both doses (1 *μ*g/mL; 10 *μ*g/mL) resulted in significant (*P* < 0.05) decrease in progesterone secretion (Figure 2). Adiponectin at any dose did not affect P_4_ secretion by luteal cells collected on days 2-3 and 14–16 (data not shown).

### 3.4. Effects of Adiponectin on Progesterone and Oestradiol Secretion by Follicular Granulosa Cells

**Figure 3:**
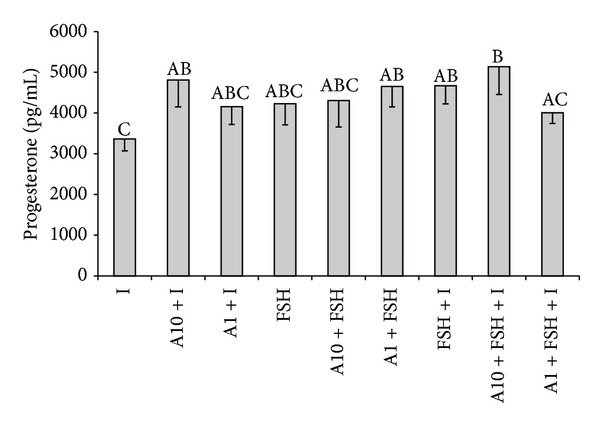
The effect of adiponectin (10 *μ*g/mL-A10 and 1 *μ*g/mL-A1) on insulin-stimulated (I; 10 ng/mL) and/or FSH-stimulated (10 ng/mL) progesterone secretion by cultured granulosa cells collected on days 17–19 of the oestrous cycle. Bars with different superscripts are significantly different (*P* < 0.05). Results are means ± S.E.M. of five replicates.

Adiponectin at the higher dose (10 *μ*g/mL) in combination with insulin provoked the increase in P_4_ secretion in comparison with the cells stimulated by insulin alone (*P* < 0.05). In contrast, there was no effect of adiponectin (both doses) on FSH- and FSH + insulin-induced production of P_4_ in porcine granulosa cells (Figure 3). Basal secretion of P_4_ was unaffected independently on adiponectin dose (data not shown).

**Figure 4:**
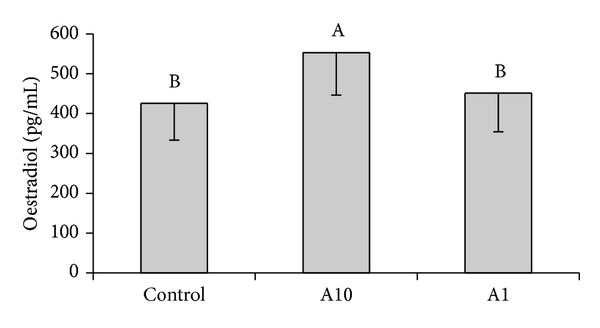
The effect of adiponectin (10 *μ*g/mL-A10 and 1 *μ*g/mL-A1) on basal oestradiol secretion by cultured granulosa cells collected on days 17–19 of the oestrous cycle. Bars with different superscripts are significantly different (*P* < 0.05). Results are means ± S.E.M. of five replicates.

Adiponectin at the higher dose (10 *μ*g/mL) increased basal secretion of E_2_ (*P* < 0.05) (Figure 4). Adiponectin did not change secretion of E_2_ by granulosa cells cultured with insulin and/or FSH (data not shown).

### 3.5. Effects of Adiponectin on Progesterone, Oestradiol, Androstenedione, and Testosterone Secretion by Follicular Theca Interna Cells

**Figure 5:**
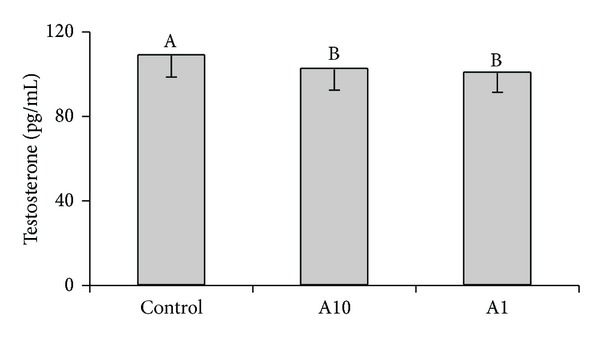
The effect of adiponectin (10 *μ*g/mL-A10 and 1 *μ*g/mL-A1) on basal testosterone secretion by cultured theca interna cells collected on days 17–19 of the oestrous cycle. Bars with different superscripts are significantly different (*P* < 0.05). Results are means ± S.E.M. of five replicates.

Adiponectin at both doses reduced basal secretion of T by porcine theca interna cells (*P* < 0.05) (Figure 5). The effect of adiponectin on basal P_4_, E_2,_ and A_4_ release was negligible (data not shown).

**Figure 6:**
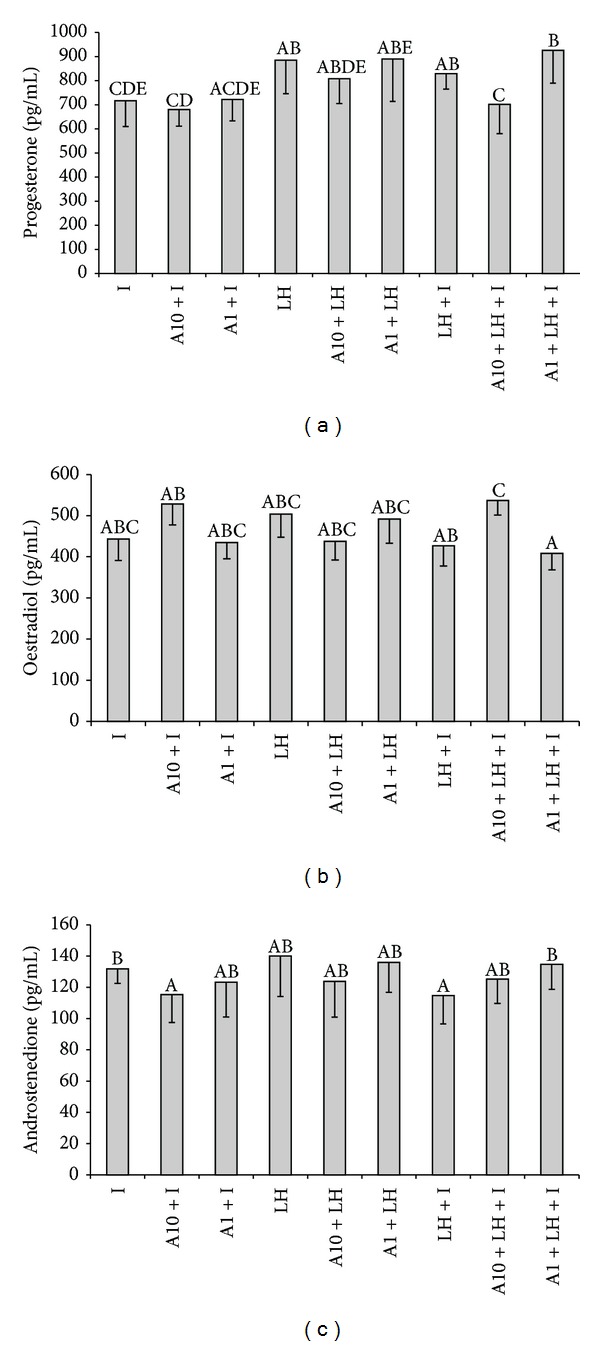
The effect of adiponectin (10 *μ*g/mL-A10 and 1 *μ*g/mL-A1) on insulin-stimulated (I; 10 ng/mL) and/or LH-stimulated (10 ng/mL) (a) progesterone, (b) oestradiol, and (c) androstenedione secretion by cultured theca interna cells collected on days 17–19 of the oestrous cycle. Bars with different superscripts are significantly different (*P* < 0.05). Results are means ± S.E.M. of five replicates.

Adiponectin (10 *μ*g/mL) decreased LH + insulin-stimulated secretion of P_4_ by theca interna cells (*P* < 0.05). There was no effect of adiponectin on LH- or insulin-induced production of P_4_ by these cells (Figure 6(a)).

Adiponectin did not influence LH- or insulin-stimulated secretion of E_2_ by theca interna cells. However, LH + insulin-induced secretion of E_2_ by the cells was increased in response to the higher dose (10 *μ*g/mL) of adiponectin (*P* < 0.05) (Figure 6(b)).

Secretion of A_4_ by theca interna cells was inhibited by combination of adiponectin (10 *μ*g/mL) with insulin compared to insulin alone (*P* < 0.05). There was no effect of adiponectin on LH-induced production of A_4_. Adiponectin at the lower dose (1 *μ*g/mL) increased LH + insulin-induced A_4_ secretion by the cells (*P* < 0.05) (Figure 6(c)). Induced secretion of T was not changed under adiponectin influence (data not shown).

## 4. Discussion

This is the first ever study to demonstrate the changes in adiponectin gene and protein expression in the porcine ovary throughout the oestrous cycle. Our results indicate that adiponectin is more highly expressed in luteal cells derived from ovaries in all examined stages of the luteal phase than in follicular granulosa and theca interna cells. Adiponectin was also found to affect basal and stimulated secretion of steroid hormones by porcine ovarian cells.

In addition to porcine ovarian structures, adiponectin gene expression and the presence of the hormone protein were also found in woman theca interna cells [16] and in theca interna cells, granulosa cells, corpora lutea, and oocytes of rats [17] and cows [18, 19]. Singh and Krishna [41] localized the adiponectin protein in bat ovarian structures that showed positive immunostaining in theca interna cells, oocytes of growing follicles, and the corpus luteum. The expression of adiponectin gene and protein was also determined in porcine granulosa cells of prepubertal gilts [8]. The levels of adiponectin expression and also its receptors seem to vary subject to cell type and the stage of follicle and corpora lutea development. In the bovine ovary, the expression of adiponectin and its both receptors was lower in granulosa cells than in theca interna cells and oocytes [18]. Contrary results were presented by Ortega et al. [42] who observed the highest levels of adiponectin expression in granulosa cells of sheep ovaries. Throughout follicular development, adiponectin mRNA increased in granulosa cells and decreased in theca interna cells of cows. In the corpora lutea, the discussed hormone was most highly expressed during luteolysis. Bovine theca interna cells derived from large follicles revealed higher expression of both receptors than cells isolated from medium-sized and small follicles, indicating that follicular growth influences the transcript levels of adiponectin receptors [18].

The synthesis of ovarian adiponectin and the expression of AdipoR1 and AdipoR2 are probably hormonally controlled, as suggested by an increase in adiponectin concentrations in ovarian follicular fluid of women administrated LH in the* in vitro* fertilization procedure [43]. Following PMSG pretreatment, an injection of hCG also significantly increased the expression of adiponectin and AdipoR1 (but not AdipoR2) genes in rat ovaries [17]. In bovine theca interna cells, LH increased the concentrations of AdipoR2 mRNAs, whereas IGF-I suppressed the expression of the above gene [44]. The results of the present study seem to confirm the hypothesis that adiponectin production is regulated hormonally. Higher levels of adiponectin gene and protein expression during the luteal phase and lower expression levels during the follicular phase could point to the stimulatory effect of progesterone and the inhibitory influence of oestradiol on ovarian adiponectin production.

In our previous study, adiponectin serum concentrations were higher during the luteal phase than the follicular phase [22], which suggests that ovarian steroids influence plasma adiponectin levels. In this study, we examined the effect of adiponectin* in vitro* on steroid secretion. The results of our* in vitro* studies and the presence of both adiponectin receptors in porcine ovaries indicate that adiponectin may directly affect ovarian steroidogenesis. In fact, progesterone secretion decreased due to adiponectin's influence on luteal cells in mid-luteal phase (days 10–12). Greater variations in adiponectin action were observed in porcine follicular cells. Induced progesterone production increased in granulosa cells and decreased in theca interna cells. Basal testosterone output and insulin-induced androstenedione secretion were inhibited, while LH + insulin-stimulated release of androstenedione was enhanced by adiponectin. The effect of adiponectin on ovarian steroidogenesis was also suggested in studies of cows, rats, and humans. Adiponectin was found to inhibit insulin-induced secretion of progesterone and oestradiol by bovine granulosa cells [19]. In a study of theca interna cells of bovine ovaries, Lagaly et al. [44] observed that adiponectin decreased LH + insulin-induced production of progesterone. The above authors also noted that adiponectin inhibited the mRNA expression of LH receptor in granulosa cells. Granulosa cells of rats and women treated with IGF-I responded to adiponectin by increasing progesterone and oestradiol secretion [16, 17]. In the cited studies, adiponectin did not influence FSH-induced production of progesterone and oestradiol in human and rat granulosa cells which is consistent with the response of porcine cells noted in this study.

It seems that adiponectin affects ovarian functions by binding AdipoR1 and AdipoR2, in two ways. An experiment where RNAi was used to block AdipoR1 and AdipoR2 expression in the KGN cell line derived from human granulosa cells contributed to new information about adiponectin's role in the ovaries. The absence of AdipoR1 in the analysed cells enhanced apoptosis, whereas the elimination of AdipoR2 reduced FSH- and IGF-I-induced the production of progesterone and oestradiol and inhibited mitogen-activated kinase activity, relative to control, in response to adiponectin or FSH treatment [45]. The above results suggest that AdipoR1 is more involved in the survival of granulosa cells, whereas AdipoR2 regulates steroidogenesis through MAPK activation. The influence of adiponectin on the ovaries of sexually mature pigs has not been studied to date, but the response of granulosa cells derived from prepubertal gilts to the analysed hormone was described by Ledoux et al. [46]. The above authors observed higher levels of cyclooxygenase-2, prostaglandin E synthase, and VEGF gene expression in cells primed with adiponectin, an increase in the expression of the StAR gene and a decrease in the expression of the P450 aromatase gene. Adiponectin's impact on steroidogenesis was also investigated in very distant species. Singh and Krishna [47] documented the influence of adiponectin in vespertilionid bats. Adiponectin treatment contributed to an increase in progesterone levels and a decrease in androstenedione and oestradiol plasma concentrations in comparison with control bats. In the same study, adiponectin treatment increased P450_SCC_ and 3*β*-HSD enzyme levels but decreased aromatase, StAR and LH-R levels in comparison with controls.

The involvement of adiponectin in steroidogenesis control is not restricted to the ovaries. Its activity was also noted in the testes and adrenal glands. An* in vitro* study by Caminos et al. [48] demonstrated that adiponectin significantly inhibited basal and hCG-stimulated testosterone secretion by testicular explants. In another study, adiponectin decreased corticosterone secretion by freshly isolated rat adrenocortical cells but did not affect aldosterone production [49]. An opposite effect was observed in human adrenocortical H295R cells. Adiponectin treatment enhanced cortisol secretion, which was accompanied by increased expression of steroidogenic pathway genes, including StAR protein expression, via ERK1/2 and AMPK-dependent pathways [50].

In addition to its direct effects on steroidogenesis in the ovary, adiponectin could also indirectly affect gonadal functions by controlling the secretory activity of the hypothalamus and pituitary. A study by Wen et al. [51] demonstrated the inhibition of GnRH release from GT1-7 hypothalamic GnRH neurons after the administration of adiponectin. In* in vivo* study by Cheng et al. [52] adiponectin also lowered GnRH secretion by activating the AMPK and inhibiting the ERK pathways. In the lower branch of the HPG axis, adiponectin exhibited a general inhibitory effect on gonadotrophins secretion. The release of LH from rat pituitary cells and the murine L*β*T2 cell line was decreased after the administration of adiponectin alone and in combination with GnRH [53, 54].* In vivo* observations also demonstrated an inhibitory effect of adiponectin on LH secretion: the intravenous administration of the adenovirus expressing the adiponectin gene to male mice decreased plasma LH levels without changes in FSH levels [54]. The inhibitory influence of adiponectin on LH secretion could be attributed to a decrease in GnRH receptor gene expression in the pituitary [53].

## 5. Conclusions

The presence of adiponectin mRNA and protein in porcine ovaries observed in this study as well as the presence of adiponectin receptors 1 and 2 in the gonads noted in our previous work could provide evidence for auto/paracrine actions of the analysed hormone. The variations in adiponectin gene and protein expression during the oestrous cycle indicate that adiponectin expression is determined by the hormonal status of pigs. The effect of adiponectin on ovarian steroidogenesis suggests that this adipokine influences reproductive functions in pigs. Yet for definitive conclusions to be drawn, especially concerning precise localization of adiponectin mRNA and protein in different ovarian structures, intracellular mechanisms of adiponectin influence on the gonadal steroidogenesis and its possible effect on other functions of the ovaries, further studies are required to determine the role of adiponectin in ovarian physiology.
